# Parametric Analysis of Failure Loads of Masonry Textures by Means of Discontinuity Layout Optimization (DLO)

**DOI:** 10.3390/ma15103691

**Published:** 2022-05-21

**Authors:** Mattia Schiantella, Federico Cluni, Vittorio Gusella

**Affiliations:** Department of Civil and Environmental Engineering, University of Perugia, via G. Duranti 93, 06125 Perugia, Italy; mattia.schiantella@studenti.unipg.it (M.S.); vittorio.gusella@unipg.it (V.G.)

**Keywords:** discontinuity layout optimization, masonry textures, upper bound limit analysis

## Abstract

Several masonry structures of cultural and historical interest are made with a non-periodic masonry material. In the case of periodic textures, several methods are available to estimate the strength of the masonry; however, in the case of non-periodic masonry, few methods are available, and they are frequently difficult to use. In the present paper we propose using discontinuity layout optimization (DLO) to estimate the failure load and mechanism of a masonry wall made with non-periodic texture. We developed a parametric analysis to account for the main features involved in the estimation of failure: in particular we considered three different textures (periodic, quasi-periodic, and chaotic), variable height-to-width ratio of the wall (from 0 to 3) and of the blocks (from 0.25 to 1), different mechanical properties of mortar joints and blocks, and possible presence of a load on the top. The results highlight the importance of the parameters considered in the analysis, both on the values of the failure load and on the failure mechanism. Therefore, it is found that DLO can be an useful and affordable method in order to assess the mechanical strength of masonry wall made with non-periodic textures.

## 1. Introduction

**Figure 1:**
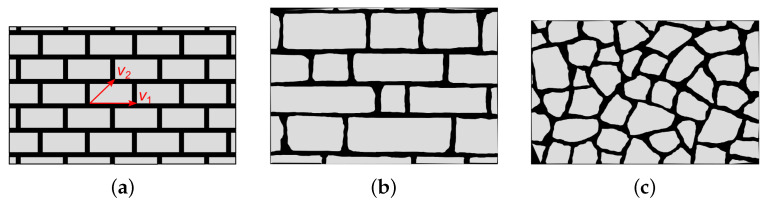
Masonry texture classification: (**a**) periodic, (**b**) quasi-periodic, (**c**) chaotic.

The great majority of historical constructions are made of masonry. Whereas for some prestigious buildings the texture of the masonry is periodic, this is not true in most cases. We define a texture as periodic if it is characterized by blocks having equal size and placed in a regular pattern, as shown in Figure 1a, so that the position of each block is given by a linear combination of two vectors (indicated as $v_{1}$ and $v_{2}$ in the figure).

When dealing with non-periodic masonry textures, we define two different typologies: the first one is the quasi-periodic texture [1], given by blocks of different sizes but arranged in such a way that we can identify masonry courses, i.e., the horizontal mortar joints are almost aligned, as can be seen in Figure 1b. It is worth noting that, as in the periodic texture, the blocks have almost a rectangular shape. The second typology is the chaotic texture, when blocks of different sizes may be arranged arbitrarily; moreover, the blocks may have a shape very far from rectangular (see Figure 1c).

In actual realizations, things may be even more complex, as in several cases the building has a multi-leaf masonry, with the exterior curtain characterized by periodic texture, the inner leaf characterized by quasi-periodic or chaotic texture, and the two leaves connected by means of a filling of (generally) poor quality [2,3]. This multi-leaf masonry will not be considered in the present paper.

Several methods have been proposed in literature for evaluating the mechanical characteristics of periodic masonry, especially for estimating the failure load. Luciano and Sacco [4] proposed a micro-mechanical approach employing a damage model suitable for old masonries; Milani [5] used an approach where the nonlinearities are in prefixed positions inside the bricks and at joints; Baraldi and Cecchi [6] proposed a method based on rigid blocks connected by joints modelled as interfaces. In [7], an approach that uses upper bound limit analysis combined with non-linear dynamic analysis (a rocking analysis) was proposed. A general approach based on finite element method can be used [8,9]. When dealing with masonry wall, the effect of load direction on shear strength can be of importance [10], and of course the orthotropic nature of the material should be taken into account [11]. A comparison of several methods to assess the response of plane masonry is reported in [12], whereas a comprehensive review of the numerical models available for structures made with (periodic) masonry is in [13], and an overview of available design method is in [14].

When dealing with non-periodic masonry, the available methods are somewhat more limited. An approach could consist in considering the actual quasi-periodic texture at the micro-scale and, extending the method used in elasticity [15,16], estimate a hierarchy of lower- and upper-bounds of failure surface using both essential and natural boundary conditions [17]. The effect of random variation of elastic modulus on a prototype of non-periodic structure has also been analyzed [18,19,20]. A different approach consists of defining a statistically equivalent periodic unit cell [21] and thereafter estimating the failure surface using one of the methods available in literature and cited previously.

Because the failure load is sought, the approach based on limit analysis is appealing. Moreover, one can think to the problem of finding the failure mechanism of the masonry as a layout optimization problem, i.e., to find the pattern of the discontinuities giving the minimum failure mechanism associated with the minimum load, in a typical upper bound estimation by means of limit analysis. This approach was proposed for the continuum as discontinuity layout optimization (DLO) in the work of Smith and Gilbert [22], and it can be considered as an alternative method to other ones available in literature, e.g., some consider directly the particles in the terrain and their interactions [23] and belong to the family of discrete element methods (DEM) [24,25]; others use the finite element method, which could require a change in the mesh to account for failure zone and increase the accuracy (an example for a slightly different field is in [26]). In DLO, the approach pioneered by Heyman [27] and subsequently used by many others (see for example [28,29], both considering periodic masonries with potential failure surfaces in the joints and inside the bricks) is adopted, and it consists of considering a great number of potential discontinuities that can appear in the continuum and of estimating the effective configuration of discontinuities at failure by minimizing a suitable function (usually the energy dissipated). The problem is closely related to finding the optimal configuration of a lattice structure by means of solving a linear programming problem [30] based on work done by [31,32]. Linear programming is also used in estimating the failure load of masonry made of rigid blocks considering non-associative friction [33]. The DLO has been subsequently applied to masonry wall [34], to retaining walls [35], in geotechnics [36], and to the yield-line method for slabs [37], and several examples of application to different civil engineering problems can be found in [38]. The computational efficiency of a discrete approach to layout optimization is shown in some recent papers [39,40]. Moreover, the DLO has been applied to steel connections [41] and used to calculate foundation over volcanic rock with a low density [42].

Because with DLO it is possible to take into account the whole masonry panel, when dealing with non-periodic textures it is a more suitable approach than other limit analysis ones, which usually require a representative (periodic) volume element.

In the present paper, the results obtained in applying DLO to non-periodic masonries are presented. We assumed that the discontinuities can appear only in the mortar joints, as usually in historic masonries the mortar is much weaker than the blocks (stones or bricks). In particular, a Mohr–Coulomb failure criterion has been used.

We used a DLO formulation already present in literature, enhancing it and modifying several aspects related to the automatic generation of the required texture in order to apply it systematically to masonry structures. As far as we know, this is the first time that this approach has been used to address a systematic analysis of non-periodic masonry and the mechanical properties of mortar and blocks. Moreover, the script has been coded from the scratch in a Matlab environment [43] and validated by comparison with existing cases previously cited.

The paper is organized as follows. In Section 2.1 the main ideas of the DLO are briefly recalled. In Section 2.2 the application to masonry with rigid block is introduced. In Section 2.3 we report the definition of the different masonry textures used. Section 3 is devoted to presenting the obtained results. In Section 3.1 the influence of cohesion is analyzed, and in Section 3.2 the effect of different height to width of the block ratio is shown. In Section 3.3 a tentative approach to take into account the possibility of discontinuities inside the blocks is presented. Some conclusions are drawn in Section 4.

## 2. Materials and Methods

### 2.1. The Discontinuity Layout Optimization (DLO)

Discontinuity layout optimization (DLO) is a procedure that was proposed in [22] for the analysis of soils in plane strain conditions, but it can be and has been adopted for this kind of analysis, as the existence of stress components normal to the surface does not influence the outcome for this limit analysis. In the present section, we recall the basic concepts presented in [22].

First of all, the domain is discretised using a finite number of points that represent the extremity of each potential discontinuity. To obtain the entire set of potential discontinuities, each point is connected to the others. An upper bound limit analysis then identifies the pattern of discontinuity at failure; this obtains the minimization of a suitable function related to the energy associated to the failure mechanism—in the present case, the total internal energy dissipated in the discontinuities, *E*. Because an upper bound approach is applied, the problem is ruled by the velocities at the discontinuities. Moreover, a set of equations is necessary to establish the compatibility at each node. In this paper, a Mohr–Coulomb failure criterion is adopted, characterized by dilation, so the compatibility conditions may be written as follows:

(1) $$\begin{array}{c} \sum_{i=1}^{n}\cos\theta_{i}\cdot s_{i}-\sin\theta_{i}\cdot n_{i}=u^{x} \end{array}$$

(2) $$\begin{array}{c} \sum_{i=1}^{n}\sin\theta_{i}\cdot s_{i}+\cos\theta_{i}\cdot n_{i}=u^{y} \end{array}$$

**Figure 2:**
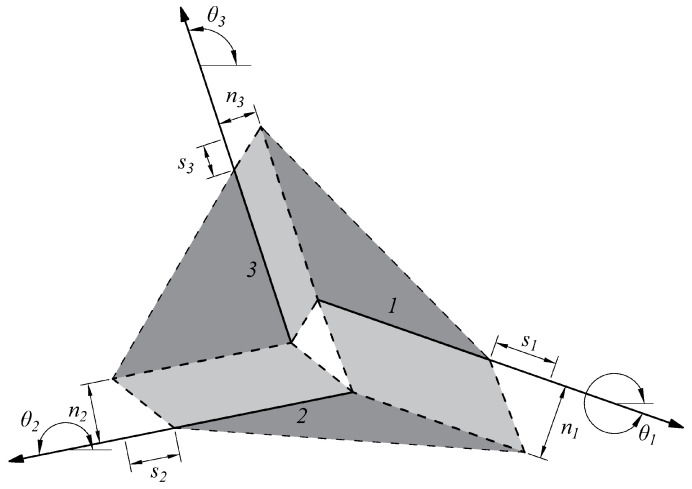
Compatibility conditions at nodes.

In these equations, *n* stands for the number of discontinuities, whereas $\theta_{i}$ is the angle of the discontinuity with respect to reference frame *x* (see Figure 2). Finally, $s_{i}$ is the velocity parallel to the discontinuity and $n_{i}$ is the normal one, and $u^{x}$ and $u^{y}$ are the nodal velocity components.

The compatibility conditions can be expressed for each discontinuity in a matrix form: $s_{i}$ and $n_{i}$ are collected in the vector $d_{i}$ and then related to the velocity of the points at the beginning and at the end of discontinuity, $u_{i}$ through the compatibility matrix $B_{i}$ as in

(3) $$B_{i}d_{i}=u_{i}$$

or in an expanded form as

(4) $$B_{i}d_{i}=\left[\begin{array}{cc} \cos\theta_{i} & -\sin\theta_{i}\\ \sin\theta_{i} & \cos\theta_{i}\\ -\cos\theta_{i} & \sin\theta_{i}\\ -\sin\theta_{i} & -\cos\theta_{i} \end{array}\right]\left[\begin{array}{c} s_{i}\\ n_{i} \end{array}\right]=\left[\begin{array}{c} {u_{A}}_{i}^{x}\\ {u_{A}}_{i}^{y}\\ {u_{B}}_{i}^{x}\\ {u_{B}}_{i}^{y} \end{array}\right]$$

the vector d containing the velocities for all discontinuities has the following form:

(5) $$d^{T}=\left[s_{1},n_{1},s_{2},n_{2},\ldots \right]$$

The power dissipated in the discontinuities *E* can be expressed by the function

(6) $$E=g^{T}\cdot d$$

Because we are minimizing *E*, we are also minimizing the work done by the external loads. If both live loads, $f_{L}$ and dead loads, $f_{D}$, are present, we have:

(7) $$E=\lambda f_{L}^{T}d+f_{D}^{T}d$$

where

(8) $$\begin{array}{c} f_{L}^{T}=\left\{f_{L1}^{s},f_{L1}^{n},f_{L2}^{s},f_{L2}^{n},\ldots ,f_{Lm}^{n}\right\} \end{array}$$

(9) $$\begin{array}{c} f_{D}^{T}=\left\{f_{D1}^{s},f_{D1}^{n},f_{D2}^{s},f_{D2}^{n},\ldots ,f_{Dm}^{n}\right\} \end{array}$$

and so the problem becomes, for the case of *m* potential discontinuities and *n* nodes and assuming a Mohr–Coulomb criterion with friction angle φ:

(10) $$\min\left\{\lambda f_{L}^{T}d\right\}=-f_{D}^{T}\cdot d+g^{T}\cdot p$$

subjected to

(11) $$\begin{array}{c} Bd=0 \end{array}$$

(12) $$\begin{array}{c} Np-d=0 \end{array}$$

(13) $$\begin{array}{c} f_{L}^{T}d=1 \end{array}$$

(14) $$\begin{array}{c} p\geq 0 \end{array}$$

with

(15) $$\begin{array}{c} g^{T}=\left\{c_{1}l_{1},c_{1}l_{1},c_{2}l_{2},c_{2}l_{2},\ldots ,c_{m}l_{m}\right\} \end{array}$$

(16) $$\begin{array}{c} N_{i}p_{i}-d_{i}=\left[\begin{array}{cc} 1 & -1\\ \tan\varphi & \tan\varphi \end{array}\right]\left[\begin{array}{c} p_{i}^{1}\\ p_{i}^{2} \end{array}\right]-\left[\begin{array}{c} s_{i}\\ n_{i} \end{array}\right]=0\;i=1,2,\ldots ,m \end{array}$$

where *p* is a 2m vector of plastic multiplier.

The preceding is a linear programming problem with variables d and p.

### 2.2. Extension to Rigid Rotations

In order to apply discontinuity layout optimization to the analysis of a masonry subjected to a seismic action, the previous formulation needs to be modified as proposed in [34], and the main aspects of the formulation are here resumed. Along with normal and shear displacements, rotations must also be considered and so d and g vectors become:

(17) $$\begin{array}{c} d^{T}=\left[s_{1},n_{1},\omega_{1},s_{2},n_{2},\omega_{2},\ldots \right] \end{array}$$

(18) $$\begin{array}{c} g^{T}=\left\{c_{1}l_{1},c_{1}l_{1},0.5c_{1}l_{1}^{2}/{\tan\varphi }_{1},0.5c_{1}l_{1}^{2}/{\tan\varphi }_{1},\ldots ,0.5c_{m}l_{m}^{2}/{\tan\varphi }_{m}\right\} \end{array}$$

The compatibility conditions are also modified to consider rotations as follows:

(19) $$B_{i}d_{i}=\left[\begin{array}{ccc} \cos\theta_{i} & -\sin\theta_{i} & \frac{l_{i}\sin\theta_{i}}{2}\\ \sin\theta_{i} & \cos\theta_{i} & -\frac{l_{i}\cos\theta_{i}}{2}\\ 0 & 0 & 1\\ -\cos\theta_{i} & \sin\theta_{i} & \frac{l_{i}\sin\theta_{i}}{2}\\ -\sin\theta_{i} & -\cos\theta_{i} & -\frac{l_{i}\cos\theta_{i}}{2}\\ 0 & 0 & -1 \end{array}\right]\left[\begin{array}{c} s_{i}\\ n_{i}\\ \omega_{i} \end{array}\right]$$

The flow rule condition constraints become:

(20) $$N_{i}p_{i}-d_{i}=\left[\begin{array}{cccc} 1 & -1 & 0 & 0\\ \tan\varphi_{i} & \tan\varphi_{i} & \frac{1}{2}l_{i} & \frac{1}{2}l_{i}\\ 0 & 0 & 1 & -1 \end{array}\right]\left[\begin{array}{c} p_{i}^{1}\\ p_{i}^{2}\\ p_{i}^{3}\\ p_{i}^{4} \end{array}\right]-\left[\begin{array}{c} s_{i}\\ n_{i}\\ \omega_{i} \end{array}\right]=0$$

For the sake of clarity, it is recalled that in this application the potential discontinuities are located only along mortar joints.

### 2.3. Masonry Textures

In order to assess the influence of the masonry texture on the failure load, three different textures have been considered:

- A periodic texture, made of equal blocks arranged according to a periodic pattern, i.e., it is possible to identify two vectors through which a linear combination involving integer numbers is able to locate every block.
- A quasi-periodic texture, where the blocks can have different width and height, and they are arranged so that each block has on its left or right a block with equal height. In this way, it is possible to identify rows on the texture, and we have continuity on the horizontal mortar joints. The vertical joints between two adjacent rows should not be aligned.
- A chaotic texture, where the blocks have different width and height and are arranged without a clear pattern. It is possible that some vertical joints are aligned.

**Figure 3:**
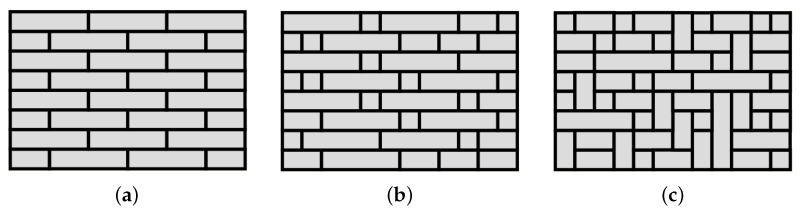
Masonry texture examples: (**a**) periodic, (**b**) quasi-periodic, (**c**) chaotic.

An example of these texture typologies is shown in Figure 3.

It is worth noting that, in the case of chaotic texture, the blocks have been reduced to rectangles in order to simplify the analysis, especially for the future realization of experimental tests. In all cases, the height of the blocks is equal, which is not true in general, but again, this is because experimental tests on sample walls, realized with half height UNI brick (with no holes) and its subdivision, are in plan [44].

## 3. Results

The discontinuity layout optimization problem has been coded from scratch in a Matlab environment [43]. An already existing case involving periodic masonry presented in [34] has been reproduced to validate the results. The latter was itself a comparison with the Ferris and Tin-Loi wall solution [45] obtained by means of MPEC.

Because it is a limit analysis, the load conditions are quasi-static and the method cannot investigate the propagation of fractures but instead provides the collapse layout of cracks associated with the given distribution of load. For the same reason, inertial effects are not considered directly but via equivalent inertial forces and moments.

Three different configurations of masonry are considered. For each of them, the variation of the load multiplier, λ, with the height/width ratio and the presence of a vertical load above the top row, *q*, is evaluated. Each elementary volume of the masonry dV is subject to a vertical body force proportional to the specific weight γ, γdV, and an horizontal body equal to λγdV. The load *q* has the dimension of a force over a surface, whereas the body force is a force per unit volume. Because this is a discrete analysis, the components for dead and live loads body forces are assigned to each potential discontinuity as follows: the weight of the strip lying above the discontinuity is evaluated by multiplying the surface for the density and then it is divided into a shear and a normal component depending on the inclination.

In the following, we assumed γ=1 and unitary thickness of the wall. It is worth noting that when q>0, a load equal to λq is applied horizontally on the top of the wall.

**Figure 4:**
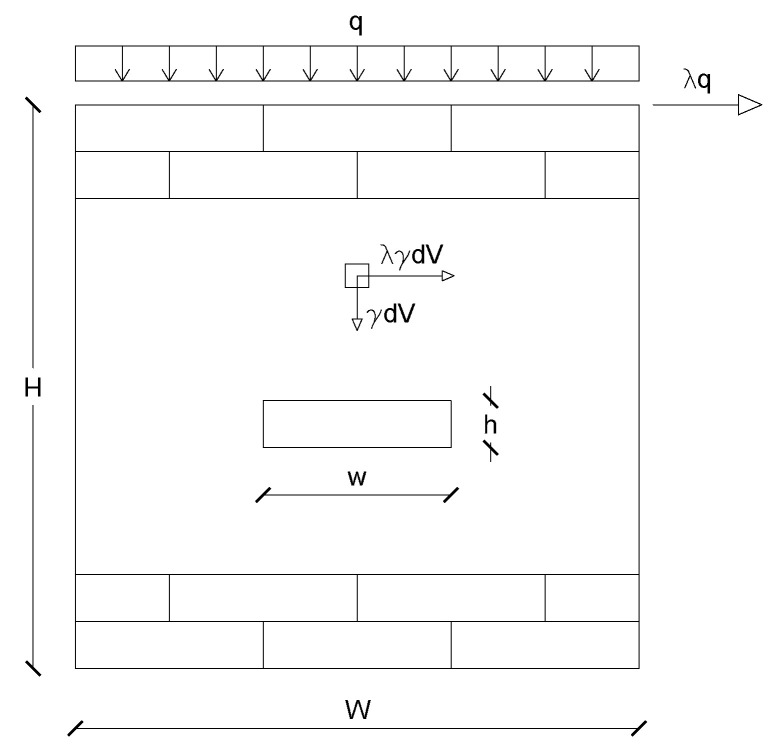
Geometrical parameters of the wall and of the blocks and applied forces.

As the aim of the parametric analysis presented in this paper is to assess the influence of the different geometrical and mechanical parameters involved in the model, the geometry of the blocks has been simplified: each block has the shorter dimension equal to 0.5 and the other one varies randomly from 0.5 to 2 by steps of 0.5. The discontinuities are considered to occur only along mortar joints, i.e., the blocks are rigid. The geometrical parameters and the forces are shown in Figure 4.

### 3.1. Lack of Cohesion

Mohr–Coulomb associative criterion is adopted, with no cohesion and coefficient of joint friction, tan(φ), taken as 0.65. The latter is a suitable value for the coefficient of friction of mortar, but similar values may be adopted without altering the following considerations on masonry textures. From the analysis we can observe the overall features of the response:

- When the height of the wall increases, the failure multiplier decreases because the failure mechanism requires less energy to activate.
- The increasing of a vertical load *q* makes the failure multiplier decreases also, due to the horizontal force associated with the load.
- For a low value of the height/width ratio, the failure multiplier is equal to tan(φ), i.e., the failure mechanism is purely translational.
- The influence of the panel texture is lower for a high H/W ratio, i.e., when H≫h and W≫w

In the following the different textures are considered in detail.

#### 3.1.1. Periodic Masonry

**Figure 5:**
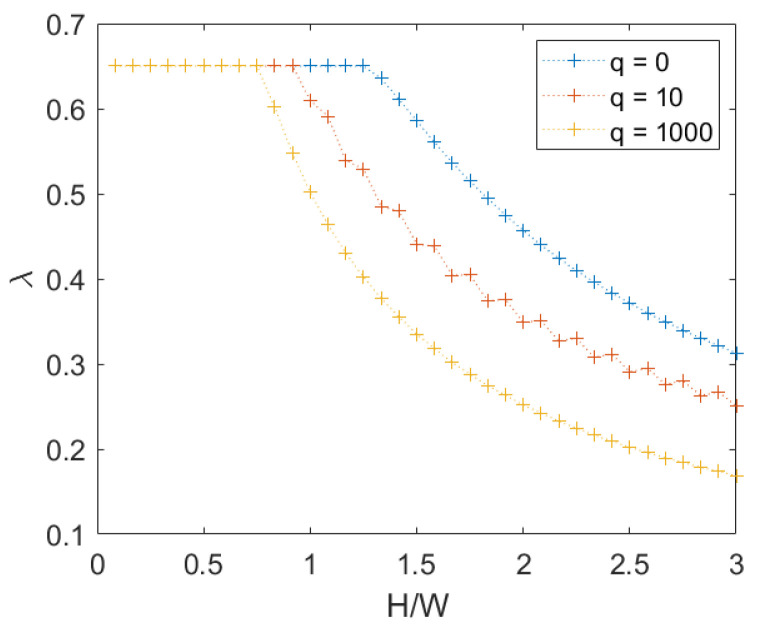
Periodic masonry: failure multipliers for different values of vertical load *q*.

We observe that for periodic masonry, as we may expect, there is a perfect symmetry of the crack pattern and the same value of the failure multiplier in both directions. When q=0, for H/W values below ≈1.25, the mechanism is translational; otherwise the mechanism is rotational, and can be also assessed with a simplified analytical calculation (see Appendix A). For greater values of *q*, the threshold between the two mechanisms reduces as *q* increases, a shown in Figure 5. It is worth noting that when q>0, the horizontal load equal to λq is applied to the top row.

**Figure 6:**
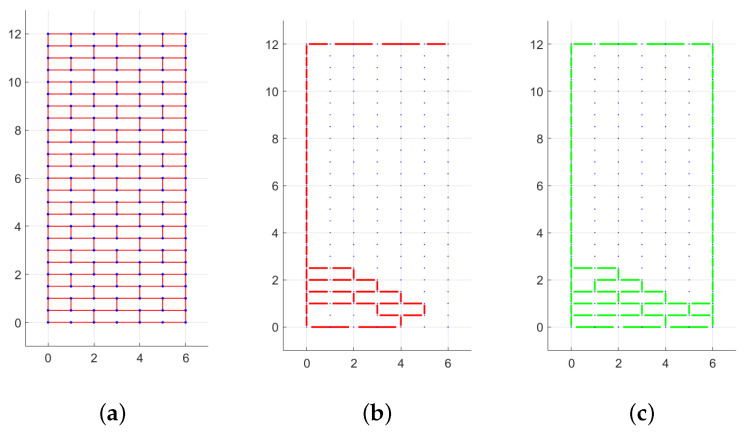
Periodic masonry with H/W=2: (**a**) texture, (**b**) mortar joints with displacements s≠0 for right oriented force, (**c**) mortar joints with rotations w≠0 for right oriented force. (Dimensions expressed in units.)

In Figure 6, the layout of mortar joints subjected to displacements and rotations for the case H/W=2 and q=0 is shown. For clarity, it is specified that in this figure and the following ones in the paper showing the texture and the failure mechanism, the axes are not labelled because the dimensions are expressed in units. The colored lines, which represent relative shear displacements and rotations, define a part of the wall that translates or rotates with respect to the other parts. In Figure 6c, for example, a great block (from height 2 to height 12) rotates clockwise with respect to the the basis, and other minor rotations are present at a lower height.

#### 3.1.2. Quasi-Periodic Masonry

**Figure 7:**
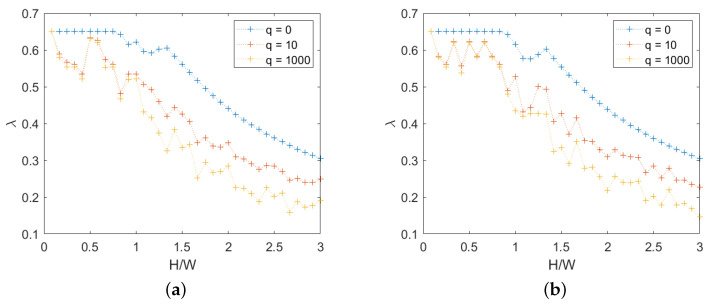
Quasi-periodic masonry, failure multipliers for different *q*: (**a**) right-oriented force, (**b**) left-oriented force.

For quasi-periodic masonry, the failure multiplier and the associated mechanism depend on direction, as shown in Figure 7.

**Figure 8:**
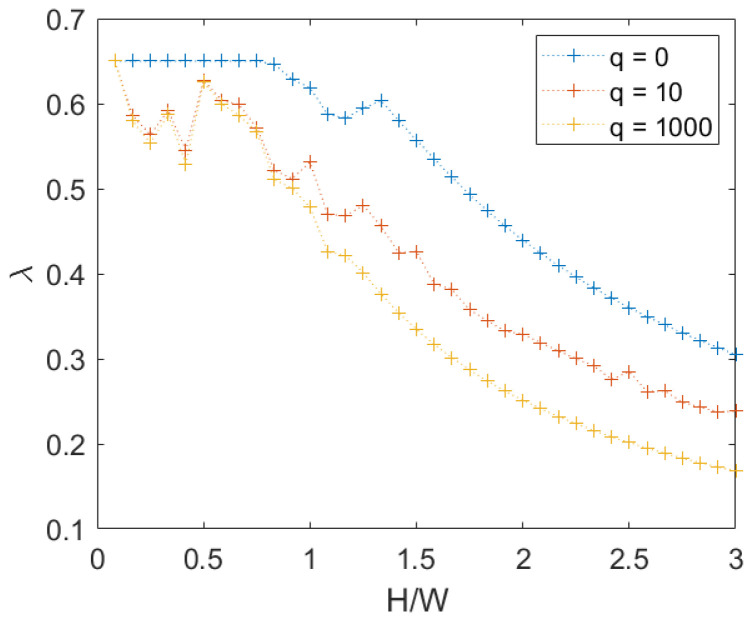
Quasi-periodic masonry, failure multipliers for different *q*: mean values for right- and left-oriented forces.

Averaging the values for right and left-oriented forces, a more regular and significant relation is obtained, as shown in Figure 8.

**Figure 9:**
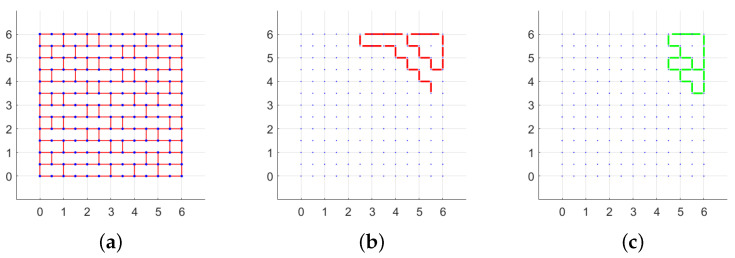
Quasi-periodic masonry with H/W=1: (**a**) texture, (**b**) layout of displacements (*s*) for right oriented force, (**c**) layout of rotations (*w*) for right oriented force. (Dimensions expressed in units.)

**Figure 10:**
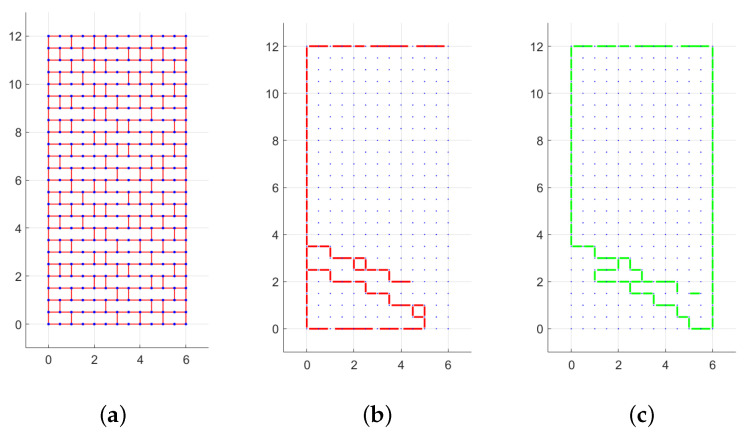
Quasi-periodic masonry with H/W=2: (**a**) texture, (**b**) layout of displacements (*s*) for right oriented force, (**c**) layout of rotations (*w*) for right oriented force. (Dimensions expressed in units.)

The transition between the pure translational mechanism and the rotational mechanism is not so neat in this case, due to the incomplete participation in rotation of the wall width. This happens for H/W between 0.8 and 1.25 in the case of q=0, and it is shown through an example of the layout of translational and rotational discontinuity for quasi-period masonry, q=0 and two different H/W ratios, respectively, equal to 1, Figure 9, and 2, Figure 10.

In this representation, only mortar joints subjected to displacements and rotations greater than 1% of the maximum values are shown. Moreover, the more irregular layout of discontinuities makes the masonry easier to be subjected to a partial failure mechanism. The latter feature is not present in the periodic masonry where the mechanism changes abruptly from pure translational to rotational. The presence of a load above the wall makes partial rotational mechanisms occur even for small *H*/*W* ratios. This is because this load has an horizontal component that is proportional to the load itself and not to the mass of the wall, and so when the *H*/*W* ratio is small, the seismic action modelled by a body force is not sufficient to cause the overturning, but the contribution of this load is significant instead. In other words, the center of the mass of the structure moves up due to the mass associated to the load.

#### 3.1.3. Chaotic Masonry

**Figure 11:**
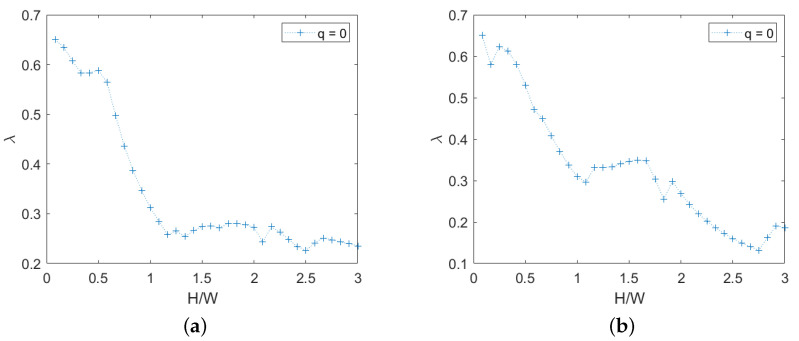
Chaotic masonry, failure multipliers for q=0: (**a**) right-oriented force, (**b**) left-oriented force.

For chaotic masonry, the failure multiplier and the associated mechanism depend on direction also, because the texture is not symmetric, as in the case of quasi- periodic texture. For this analysis, a single random wall with H/W equal to 3 has been generated, and for every smaller height the top rows have been removed, regularizing the upper boundary, presenting an irregular surface due to the presence of non-horizontal mortar planes. Only the response for q=0 is shown in Figure 11.

It may be seen that for chaotic masonry the failure multiplier is much lower, due to the presence of vertical oriented stone elements that are easier to overturn.

**Figure 12:**
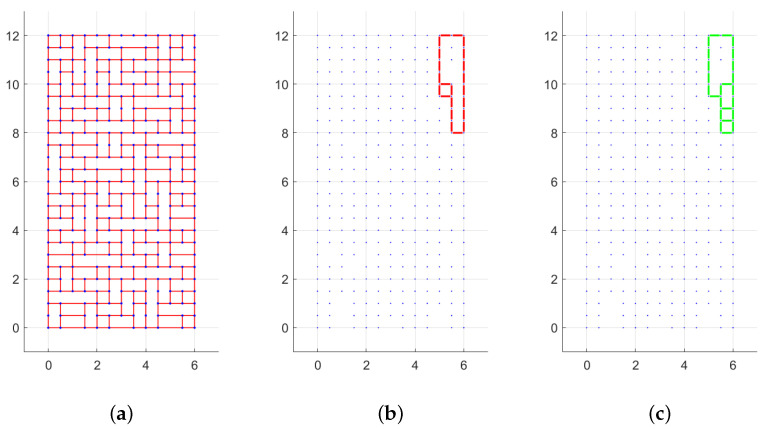
Chaotic masonry with H/W=2: (**a**) texture, (**b**) layout of displacements (*s*) for right oriented force, (**c**) layout of rotations (*w*) for right oriented force. (Dimensions expressed in units.)

For the chaotic texture, the failure mechanism involves just a part of the width of the panel, even for high values of H/W. This is shown for H/W=2 in Figure 12.

**Figure 13:**
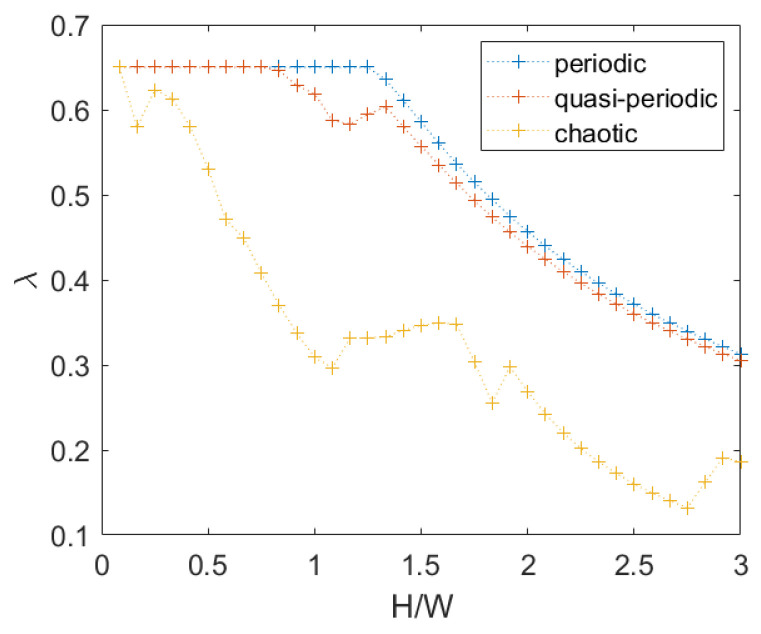
Comparison of failure multiplier at q=0 for different textures (averaging the response to left- and right-oriented forces for quasi-periodic and chaotic textures).

A comparison between the three different textures for q=0 is shown in Figure 13.

It may be observed that the effect of the difference between the periodic and quasi-periodic textures is relevant just in the transition between translation and rotation (for H/W values between 1 and 1.4), whereas for higher values of H/W, a global rotational mechanism prevails; therefore, the effect of shifting the vertical mortar joint positions can be neglected.

### 3.2. Influence of Cohesion

In the present section the influence of cohesion, *c*, in the failure criterion is investigated. In particular, in all the analyses that follow, *c* is unitary.

For the sake of simplicity, only the case of periodic masonry is shown in the following figures. When only a translational mechanism was involved, the presence of cohesion provides an increase of the failure multiplier inversely proportional to the wall height (see Appendix A).

**Figure 14:**
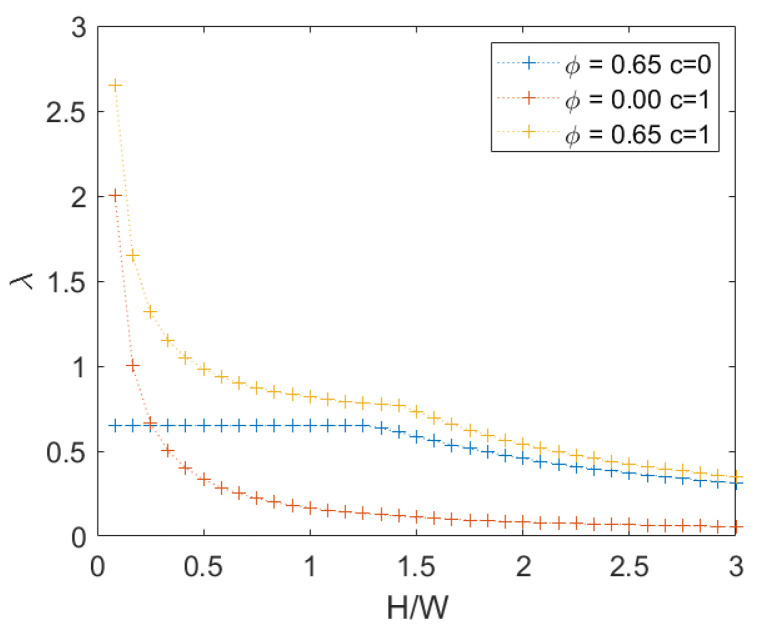
Periodic masonry: failure multiplier for different combinations of cohesion *c* and internal friction angle φ.

When a rotational mechanism occurs instead, the increase depends on the configuration at failure. In Figure 14, it is possible to observe the values of the failure multiplier for q=0. It is also possible to observe in the same figure that the influence of cohesion is negligible for an high height/width ratio, where the mechanism is rotational.

**Figure 15:**
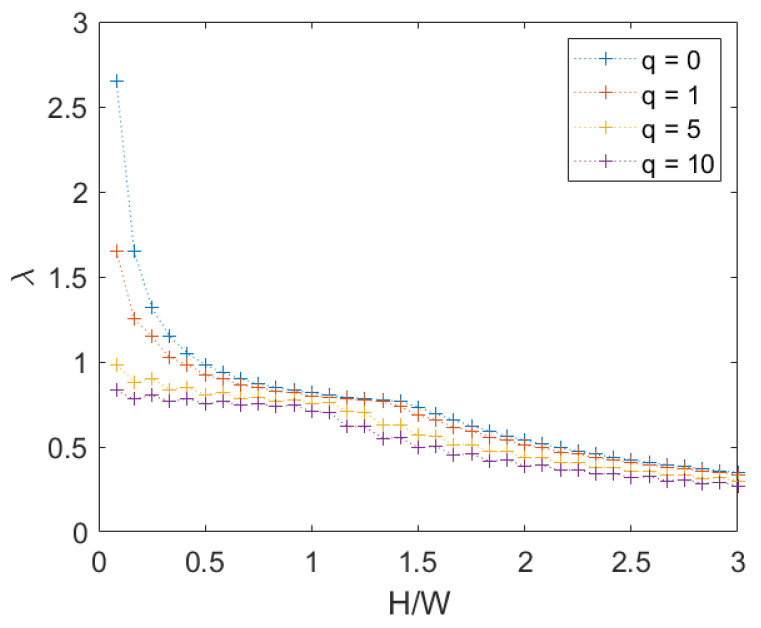
Periodic masonry: failure multiplier for c=1 and several set of loads *q*.

When the load on the top increases instead, we can see that the presence of cohesion is negligible due to the arise of a rotational mechanism even for a small height/width ratio, as explained in Section 3.1.2. This is visible in Figure 15.

### 3.3. Effect of Block Height-To-Width Ratio

**Figure 16:**
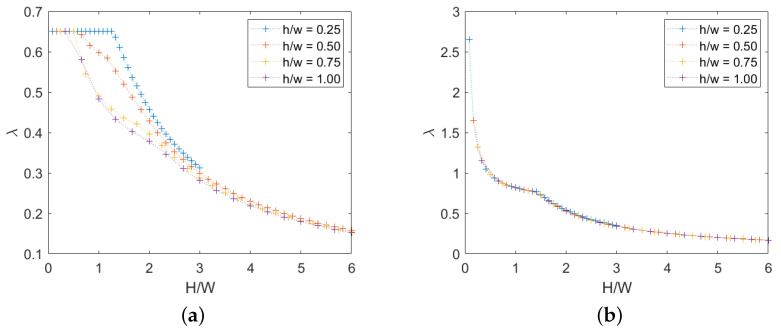
Periodic masonry: failure multiplier for different values of h/w ratio: (**a**) c=0, (**b**) c=1.

We now take in account the variation of the h/w ratio in the cases of both absence and presence of cohesion. Several ratios were investigated, with the following results. For the first case, see Figure 16a, it is observed that a higher h/w ratio makes the rotational mechanism occur for lower H/W ratio due to the tendency of slender elements to be overturned, as can also be seen in the Appendix A. When cohesion is present instead, see Figure 16b, it appears that h/w ratio does not affect the solution and the value of the H/W ratio associated with the transition from translational to rotational.

We note that the number of rows is constant and therefore the overall height of the panel increases as *h* increases.

### 3.4. Extension for Non-Rigid Stone Elements

**Figure 17:**
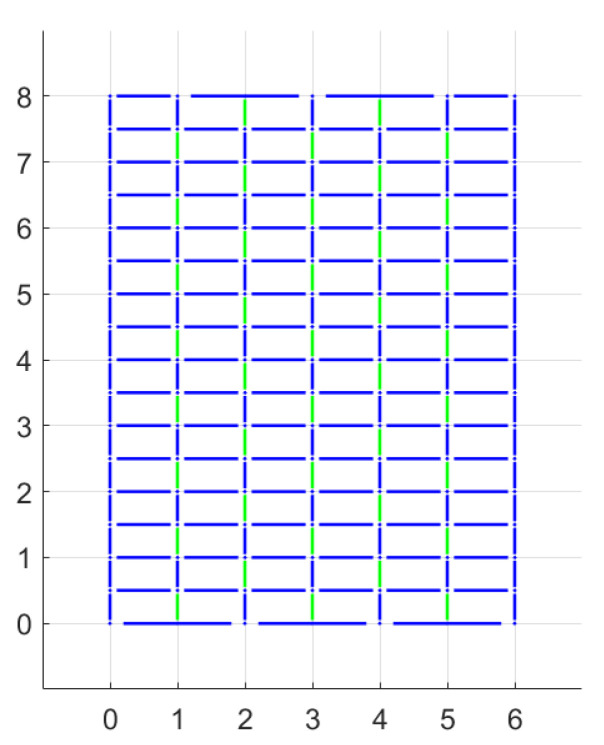
Potential discontinuities assuming non-rigid blocks: blue lines represents discontinuities belonging to mortar joints and the green ones discontinuities within the blocks. (Dimensions expressed in units.)

In all the aforementioned models, the cracks were supposed to occur only along mortar joints and therefore only the properties of mortar were considered. If we suppose that the response of the stone elements cannot be considered rigid, more potential discontinuities have to be modelled with different properties from the already present ones. For a first step, a periodic masonry is going to be analysed, and the new discontinuities will be considered to be just vertical and in the middle of the stone elements. The configuration is shown in Figure 17: blue lines represents mortar joints and the green ones the new potential discontinuities considered. A grid with joints spaced 1.0 in horizontal and 0.5 in vertical directions is used.

**Figure 18:**
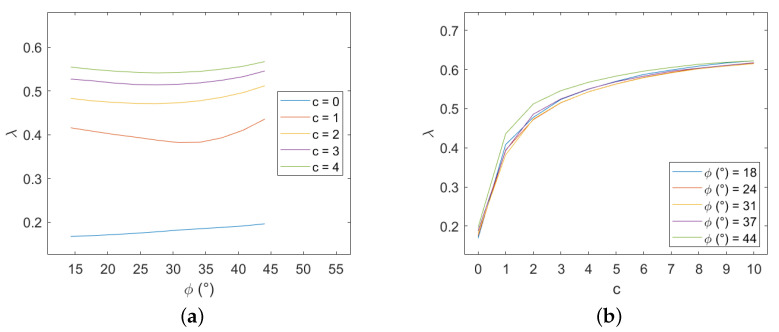
Periodic masonry, failure multiplier for different values of cohesion *c* and internal friction angle φ of the blocks: (**a**) effect of variation of φ for different values of *c*, (**b**) effect of variation of *c* for different values of φ.

A Mohr–Coulomb criterion is adopted to describe the stone elements. Several combinations of cohesion and friction for the blocks have been considered, letting the properties of the mortar constant (c=0 and tan(φ)=0.65). The variation of the failure multiplier with these two parameters has been analyzed in two different plots, as shown in Figure 18 for H/W=4/3 and q=0, and it led to some expected considerations.

First of all, the failure multiplier cannot be higher than in the previous case because adding more potential discontinuities can only result in a decrease of energy required for the mechanism activation. As we may expect, when the properties of the stone elements are far better than those of the mortar joints, the failure multiplier does not change from the previous case, and the mechanism will affect only mortar joints. The opposite is true when the properties of mortar joints are superior; the masonry panel is equivalent to a rigid stones panel with half-length stone elements and with vertical mortar joints aligned. That can bring to a relevant decrease to the failure multiplier. Between these extreme cases, the failure multiplier increases both with stone cohesion and friction as expected. Moreover the influence of cohesion variation appears higher than that of friction variation.

**Figure 19:**
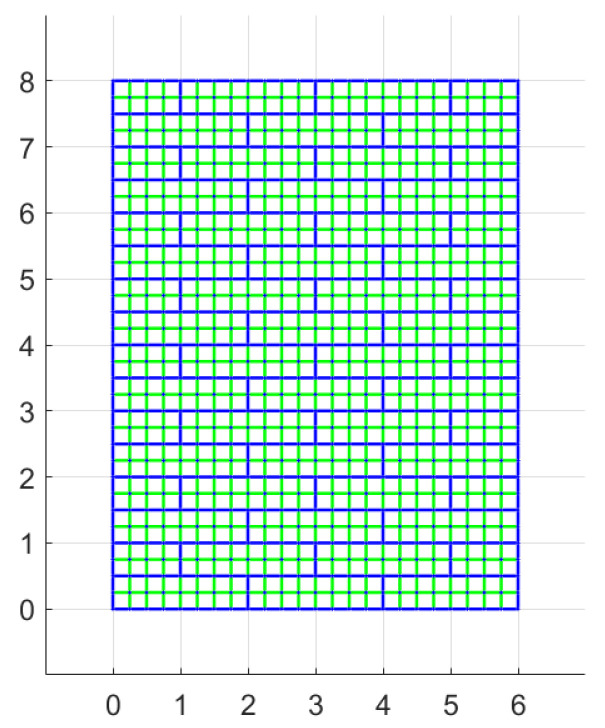
Potential discontinuities assuming non-rigid blocks with a refined discretization: blue lines represents discontinuities belonging to mortar joints and the green ones discontinuities within the blocks. (Dimensions expressed in units.)

In order to assess the influence of the mesh used to define the potential discontinuities, a refined discretization is now considered and the difference in terms of results with the latter one are discussed. In particular, a grid with dimension of 0.25 both in the horizontal and vertical directions is used, as shown in Figure 19.

**Figure 20:**
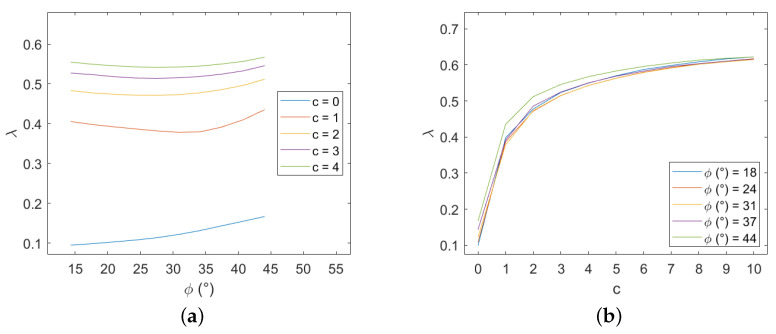
Periodic masonry, failure multiplier for different values of cohesion *c* and internal friction angle φ of the blocks: (**a**) effect of variation of φ for different values of *c*, (**b**) effect of variation of *c* for different values of φ.

This new configuration leads to results that are extremely close to the precedent discretization ones, see Figure 20, except when brick cohesion is equal to 0.

## 4. Conclusions

In the present paper, we presented some results obtained in the parametric analysis for the determination of the failure load of masonry considering different textures. The approach used was based on discontinuity layout optimization (DLO), which belongs to the family of upper bound methods of limit analysis. Three different typologies of masonry textures have been considered: periodic, quasi-periodic, and chaotic. The rules for the classification of textures are given in the text. Several parameters were considered in the parametric analysis, besides the texture itself: the effect of the height-to-width ratio of the wall; the presence of a load on the top row of the masonry panel, which contributes both to vertical load and to horizontal load; the value of the cohesion in the mortar joints; the effect of the height-to-width ratio of the blocks; the possibility that the discontinuities can appear also in the blocks.

These are the key findings regarding the study of the influence of texture:

- Only the periodic texture has a symmetric behaviour for both the failure multiplier and the failure mechanism.
- The threshold between translational mechanism and rotational mechanism when H/W varies depends on the texture: for the periodic one it is neat, for the quasi-periodic one there is a transition due to the incomplete participation to rotation of the wall width for a certain range of H/W ratios (between 0.8 and 1.25), and for the chaotic one rotations occur even for small values of H/W due to the presence of vertical elements that are easier to overturn.
- The presence of a vertical load applied on the top and its associated seismic mass leads to a decrease of the failure multiplier and it makes the rotations appear for lower H/W values.

Moreover, for a periodic texture, more studies about the mechanical property of mortar, the influence of the shape of the bricks (h/w ratio), and the possibility for the failure to occur within the blocks have been addressed, with the following results:

- The effect of cohesion in mortar joints has been studied and the results are checked by comparison with analytical formulas in the Appendix A for the case of translation.
- When h/w increases (i.e., for slender elements), the failure multiplier decreases as expected.
- If the blocks are not rigid, their mechanical properties and especially the cohesion highly influence the failure multiplier.

It is worth noting that, especially in the case of the chaotic texture, the geometry of the blocks was highly simplified; as a future step of our work, we are considering removing this limitation. Moreover, we are investigating the possible use of different failure criteria.

Although the method has been validated by comparison with other methods for the case of periodic masonry, more experimental data are needed to supplement the validity and rationality of the theoretical model for other textures and for various mechanical properties. This is an interesting development in the future research and it is already planned.

The results are interesting, and the DLO appears to be an efficient method for the failure analysis of masonry walls even with non-periodic textures, allowing the researcher to control the effects of the main parameters involved in the model in a simple and affordable way.

## Figures and Tables

**Figure A1:**
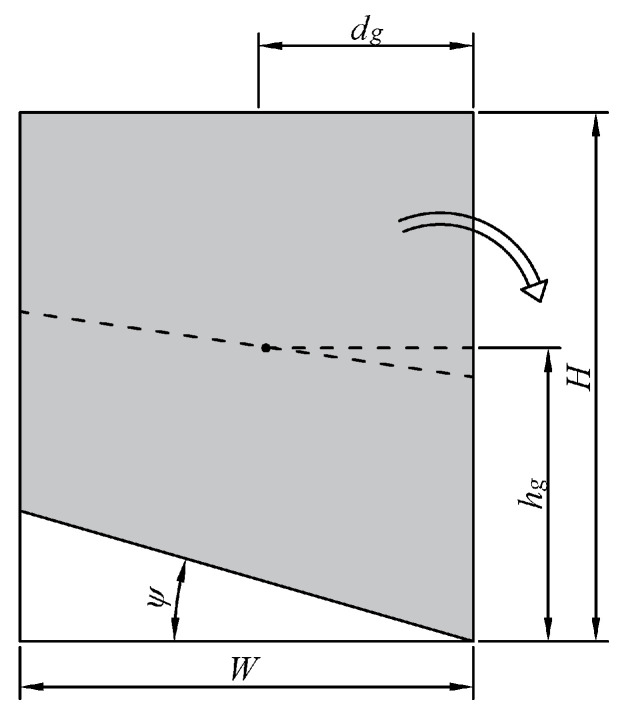
Rotation mode failure mechanism of the wall.

**Figure A2:**
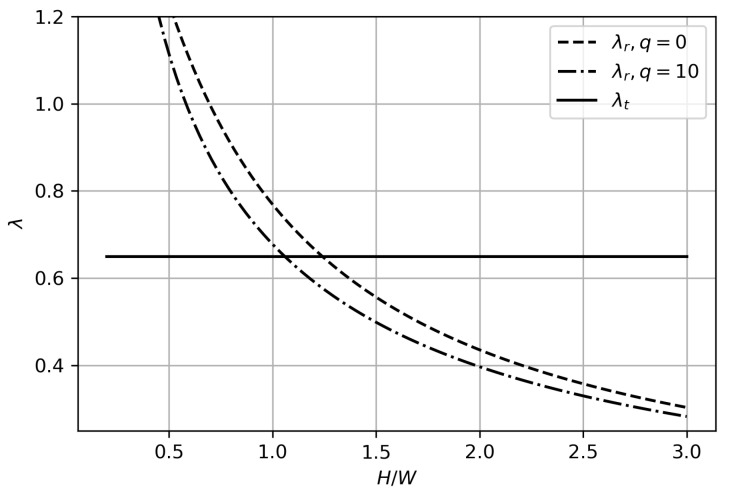
Values of $\lambda_{r}$ and $\lambda_{t}$ for various values of H/W and two different values of *q*.

## Abbreviations

The following abbreviations are used in this manuscript:

DLO	Discontinuity Layout Optimization
MPEC	Mathematical Program with Equilibrium Constraints

## Appendix A

We consider two different failure mechanisms for the wall:

- Translation of the entire wall slipping over the base;
- Rotation of a portion of the wall around one of the edges of the base.

In the rotational mechanism, it is assumed that a portion delimited by a line inclined of ψ is involved, see Figure A1.

Adopting a Mohr–Coulomb criterion, in the case of a translational mechanism we have that the value of λ at failure, $\lambda_{t}$, is given by

(A1) $$\lambda_{t}\left(\frac{\gamma WHt}{Wt}+q\right)=\left(c+\frac{\gamma WHt+qWt}{Wt}\tan\varphi \right)\Rightarrow \lambda_{t}=\frac{c+(\gamma H+q)\tan\varphi }{\gamma H+q}.$$

In the case of a rotational mechanism, we have that the value of λ at failure, $\lambda_{r}$, is given by

(A2) $$\lambda_{r}\left(\gamma Ah_{g}+qH\right)=\gamma Ad_{g}+q\frac{W}{2}\Rightarrow \lambda_{r}=\frac{\gamma Ad_{g}+q\frac{W}{2}}{\gamma Ah_{g}+qH}$$

where *A* is the area of the portion of the wall involved in the rotation and $\left(d_{h},h_{g}\right)$ locate its centroid with respect to the pivot point:

(A3) $$\begin{array}{c} A=\frac{W\left(2H-W\tan\psi \right)}{2} \end{array}$$

(A4) $$\begin{array}{c} d_{g}=\frac{W}{3}\frac{3H-W\tan\psi }{2H-W\tan\psi } \end{array}$$

(A5) $$\begin{array}{c} h_{g}=\frac{H}{2}+d_{g}\tan\psi \end{array}$$

In the case γ=1, t=1, c=0, q=0, and tanψ=h/w=1/4, we have that $\lambda_{r}=\lambda_{t}$ for H/W≈1.24, whereas with q=10 we have $\lambda_{r}=\lambda_{r}$ for H/W≈1.06, showing that as *q* increases the transition point decreases.

The values of $\lambda_{t}$ and $\lambda_{r}$ with the previously used values of γ, *t*, *q*, *c*, and tanψ are shown in Figure A2.
